# The impact of different etiologies of diminished ovarian reserve on pregnancy outcome in IVF-ET cycles

**DOI:** 10.3906/sag-1811-175

**Published:** 2019-08-08

**Authors:** Runa ÖZELÇİ, Oya ALDEMİR, Serdar DİLBAZ, Enis ÖZKAYA, İnci KAHYAOĞLU, Berna DİLBAZ, Özlem MORALOĞLU TEKİN

**Affiliations:** 1 Department of Reproductive Endocrinology and Infertility, University of Health Sciences, Etlik Zübeyde Hanım Women’s Health Training and Research Hospital, Ankara Turkey; 2 Department of Reproductive Endocrinology and Infertility, Zeynep Kamil Women’s Health Training and Research Hospital,İstanbul Turkey

**Keywords:** Poor ovarian response, IVF-ICSI, age, ovarian surgery

## Abstract

**Background/aim:**

Diminished ovarian reserve (DOR) represents a major challenge in reproductive medicine, as it is often associated with poor ovarian stimulation response, high cycle cancellation rate, and low pregnancy rate. The aim of the present study is to compare the clinical pregnancy rates in intracytoplasmic sperm injection-embryo transfer (ICSI-ET) cycles in patients with different DOR etiologies.

**Materials and methods:**

Patient data were recorded with a computer-based program called Success Estimation Using a Ranking Algorithm (SERA). Overall, 459 patients were divided into 3 groups according to their DOR etiologies (Group A: idiopathic, n = 81; Group B: age-related, n = 294; Group C: previous ovarian surgery, n = 84).

**Results:**

Out of 459 stimulation cycles, 378 (82.4%) reached the oocyte retrieval stage, while 201 (43.8%) had embryo transfers. There was no significant difference between the patients with different DOR etiologies in terms of embryo transfer and cycle cancellation rate. The patients who had embryo transfer were 44 (52.4%) in Group A, 38 (46.9%) in Group B, and 119 (40.5%) in Group C. There were no significant differences between the three groups (P = 0.114). The percentages of women who had oocyte retrieval were 84.5% in Group A, 70% in Group B, and 80.3% in Group C (P = 0.104). While clinical pregnancy per transfer was 35.8% in Group A, 19.8% in Group B, and 29.5% in Group C, there was no statistically significant difference between the groups (P = 0.113).

**Conclusion:**

Although ovulation induction and ICSI-ET outcomes, including clinical pregnancy and live birth rates, were not significantly different with regards to the etiology of DOR, young women with DOR may benefit from assisted reproductive techniques.

## 1. Introduction

Diminished ovarian reserve (DOR) represents a major challenge in reproductive medicine. The etiologic factors leading to DOR show a wide spectrum, ranging from previous ovarian surgery or chemotherapy to idiopathic premature ovarian failure. DOR is characterized by poor fertility outcomes and is often associated with a poor ovarian stimulation response, a high cancellation rate of in vitro fertilization embryo transfer (IVF-ET) cycles, and a significant decline in pregnancy rates even when assisted reproductive techniques are used [1].

Poor ovarian response (POR) to ovarian stimulation is a condition that seems to be common among women with DOR. To standardize the definition of POR, Ferraretti et al. [2] proposed new criteria, known as the Bologna criteria, based on 3 conditions: 1) advanced maternal age (>40 years) or any other POR risk factor, 2) a previous incident of POR, and 3) a low ovarian reserve test in terms of anti-Müllerian hormone (AMH) and antral follicle count (AFC). A POR diagnosis requires 2 of these 3 criteria to be met.

Although the Bologna criteria were found to be useful in predicting the outcome of IVF and for counseling purposes, their use in clinical trials has been questioned because they entail the risk of grouping together women with a large spectrum of various pathologies. 

Recently, the POSEIDON group (Patient-Oriented Strategies Encompassing Individualized Oocyte Number) proposed a new detailed stratification of low responders to ovarian stimulation from POR to a low prognosis concept. Subgroups were suggested based on quantitative and qualitative parameters: 1) age, and the expected aneuploidy rate; 2) ovarian biomarkers (AFC and AMH); and 3) ovarian response, provided that a previous stimulation cycle was performed. The new classification introduces a more nuanced picture of the “low prognosis patient” in ART, using clinically relevant criteria to guide the physician to most optimally manage this group of patients [3].

Although a consensus exists on the concept of DOR, the definition has been somewhat variable. DOR has to be distinguished from POR. As reported by Cohen et al., for the definition of DOR, any of the risk factors for POR and/or an abnormal ovarian reserve test (i.e. antral follicle count <5–7 follicles or AMH <0.5–1.1 ng/mL) could be used [1].

Although DOR has been shown to be one of the main factors that results in POR, currently there are only 2 studies comparing IVF outcomes in women with different DOR etiologies in the literature [4,5]. Thus, we do not know how different etiologies of DOR affect the clinic pregnancy rate in IVF/ICSI-ET cycles.

Identification of the differences in success rates of IVF/ICSI-ET cycles in women with different DOR etiologies might be important in individualization of the treatment protocols in order to achieve better clinical outcomes, leading to this group of women being able to avoid repetitive, prolonged, tiring, and costly procedures.

The aim of our study was to compare ICSI-ET clinical pregnancy rates among patients with different DOR etiologies (idiopathic DOR, age-related DOR, and DOR in patients with previous ovarian surgery). 

## 2. Materials and methods

Patient data were recorded with a computer-based program designed for developing a new technique called Success Estimation Using a Ranking Algorithm (SERA), using the RIMARC (ranking instances by maximizing the area under the ROC curve) algorithm [6]. The study group included 459 consecutive patients with DOR who met the inclusion criteria and were scheduled for their first ICSI-ET cycles at the Reproductive Endocrinology and Infertility Department of Etlik Zübeyde Hanım Women’s Health Training and Research Hospital between December 2010 and July 2017. 

The study was approved by the institutional ethics committee (26/06/2017/-12). As it is a retrospective study, study-specific consent was not required.

In our study, DOR was defined as having a basal FSH value of >10 IU/L and E2 of >80 pg/mL (Advia Centaur, Siemens, Germany), measured within 1–3 months of the IVF cycle, and AFC of <7 or AMH levels of <1.1 ng/mL, measured 6 months prior to the ICSI cycle. The exclusion criteria were undergoing a fertility preservation cycle, an oocyte donation cycle, or a frozen-thawing embryo transfer cycle, or having an ovarian reserve test that did not fulfill the described criteria .

Patients were divided into 3 groups: Group A, which included patients with idiopathic DOR (age <35 years and no history of ovarian surgery or chemotherapy); Group B, which included patients over 35 years of age without any surgical history; and Group C, which included patients with DOR diagnosed after ovarian surgery for endometrioma.

Patients underwent controlled ovarian stimulation according to the presentation of each case by using conventional protocols such as a flexible GnRH antagonist protocol, the GnRH agonist long protocol, or the GnRH agonist microdose flare-up protocol. 

In conventional protocols, recombinant FSH (Gonal-F, Merck Serono, Germany; Puregon, Organon, the Netherlands) with or without human menopausal gonadotropin (Menogon, Ferring Pharmaceuticals, Germany; Merional, IBSA, Switzerland) was used at doses ranging from 150 IU/day to 450 IU/day in accordance with body mass index, patient’s age, and the number of antral follicles. Patients underwent pituitary downregulation using the luteal long protocol with a GnRH agonist (Lucrin, Abbott, France) or the GnRH antagonist protocol (Cetrotide, 0.25 mg/day, Serono, Germany). The GnRH agonist microdose flare-up protocol was used with the agonist (Lucrin, Abbott, France). Subjects were monitored every 2–4 days via transvaginal ultrasound (TVS) (General Electric Logiq A5, USA) performed by a physician, starting on day 5 of the ovarian hyperstimulation, and the diameter of the ovarian follicles and the endometrial thickness were recorded. The dose of gonadotropin was adjusted according to the ovarian response. Blood samples were taken at the time of ultrasonography and serum luteinizing hormone (LH), progesterone (P), and estradiol (E2) were measured until the day of human chorionic gonadotropin (hCG) injection. A premature LH rise was defined as LH >10 IU/L, and the combination of an LH level of >10 IU and P >1 ng/mL was accepted as premature luteinization (PL). In the case of PL, hCG was administered immediately and oocyte pick-up was scheduled. When at least 3 follicles reached a mean diameter of ≥18 mm, 250 μg of recombinant human chorionic gonadotropin (Ovitrelle, Merck Serono, Germany) or 10,000 IU human chorionic gonadotropin (Pregnyl, Schering-Plough, Turkey) was administered. The oocytes were retrieved about 34 to 36 h after the hCG injection. The oocytes were inseminated by using intracytoplasmic sperm injection. The embryos were transferred 3 or 5 days after the retrieval of the oocytes, depending on the quality of the gametes. All of the subjects received luteal phase support starting on the day of oocyte retrieval by using either a daily dose of 100 mg of progesterone in oil (Progestan, Koçak, İstanbul) or vaginal progesterone (Crinone 8% gel, Merck, Germany). Luteal support was continued until the pregnancy test was performed, and in cases of pregnancy up to 10–12 weeks of gestation. In the absence of follicular growth during ovarian stimulation, the cycle was cancelled. 

Embryo transfer (ET) cancellation was defined as not having an embryo transfer due to absence of oocyte retrieval at the time of follicle aspiration, immature oocyte retrieval, abnormal fertilization, or fertilization failure leading to no embryos to be transferred.

Pregnancy was diagnosed by a positive blood test for hCG 14 days after the transfer procedure. The test was considered as positive when hCG was >10 IU/L and the test was repeated when the value was near the cut-off value. Implantation rate was defined as the percentage of embryos that successfully underwent implantation compared to the number of embryos transferred. One positive hCG result was accepted as a biochemical pregnancy. Determination of the embryonic heartbeat at TVS was defined as a clinical pregnancy. 

### 2.1. Statistical analysis

All analyses were performed SPSS 18.0 (SPSS Inc., Chicago, IL, USA). The Kolmogorov–Smirnov test was used to assess the normality of the distribution of variables. Data are presented as mean ± standard deviation. Comparisons between groups were performed using an ANOVA test. The chi-square test was used to compare pregnancy and cancellation rates. Odds ratios of each variable were calculated by using a binary logistic regression test. The enter model of multivariate regression analysis was used to calculate adjusted associations. The area under the receiver operating characteristic (ROC) curve was computed to assess the predictive accuracy of the age to predict the clinical pregnancy. P < 0.05 was considered statistically significant.

## 3. Results

Assisted reproduction cycles of 459 patients with diminished ovarian reserve were analyzed. Among these patients, 84 patients had DOR due to a previous ovarian surgery (Group A), 81 patients had early ovarian aging (idiopathic) (Group B), and 294 had age-related diminished ovarian reserve (Group C). Of the 459 cycles, 378 (82.4%) reached the oocyte retrieval stage while 201 (43.8%) had embryo transfers and 50 (10.8%) achieved a clinical pregnancy after ICSI-ET. The clinical characteristics of the IVF cycles included are shown in Table 1. There was no significant difference among the 3 groups in terms of serum AMH levels and AFC.

**Table 1:** Clinical characteristics of the in vitro fertilization cycles in women with diminished ovarian reserve.

	Group A, n = 81,mean ± SD	Group B, n = 294,mean ± SD	Group C, n = 84,mean ± SD	P-value
Age (years)	33.2 ± 1.7a	40.9 ± 2.9ab	34.71 ± 5.0b	0.040*
AMH (ng/mL)	0.43 ± 0.34	0.32 ± 0.33	0.42 ± 0.28	0.073
FSH (IU/L)	12.15 ± 9.28	12.26 ± 6.47	13.97 ± 9.54	0.176
Estradiol (pg/mL)	53.08 ± 41.18	52.75 ± 43.51	59.58 ± 59.95	0.489
Antral follicle count	3.44 ± 1.72	3.43 ± 1.59	3.67 ± 1.66	0.509
İnfertility duration (months)	77 ± 59	77 ± 82	70 ± 58	0.757
Stimulation days	10.69 ± 3.83	10.81 ± 1.66	11.30 ± 5.80	0.983
Total FSH dose (IU)	2591 ± 1767	2815 ± 1707	2977 ± 1544	0.870
Peak estradiol (pg/mL)	807 ± 861	728 ± 573	770 ± 837	0.603
Endometrial thickness on hCG day (mm)	6.8 ± 3.2	7.1 ± 3.5	8.1 ± 3.6	0.077
Number of oocyte retrieved	2.85 ± 3.54	3.41 ± 2.48	3.41 ± 2.58	0.305
Mature oocytes	2.57 ± 2.20	2.58 ± 2.41	2.59 ± 2.37	0.970
2PN	1.49 ± 1.63	1.42 ± 1.25	1.50 ± 1.56	0.734
Top quality embryo	0.60 ± 0.68	0.52 ± 0.62	0.55 ± 0.66	0.056
Number of embryos transferred	1.3 ± 0.4	1.1 ± 0.5	1.2 ± 0.3	0.089
Implantation rate	0.73 ± 0.75	0.57 ± 0.54	0.67 ± 0.74	0.195
	Idiopathic DOR(n = 81), n (%)	Age-related DOR(n = 294), n (%)	Previous ovarian surgery(n = 84), n (%)	P
Clinical pregnancy, n (%)	14 (35.8)	23 (19.8)	13 (29.5)	0.113
ET cancelled, n (%)	43 (53.1)	175 (59.5)	40 (47.6)	0.174
OPU cancelled, n (%)	10 (12.3)	58 (19.7)	13 (15.5)	0.104

Data are expressed as mean ± standard deviation or number (%); NS: not significant; DOR: diminished ovarian reserve; AMH: anti-Müllerian hormone; FSH: follicle-stimulating hormone; hCG: human chorionic gonadotropin: PN: pronuclear; ET: embryo transfer; OPU: oocyte pick-up, *: P-values with statistical significance (P < 0.05). a Pairwise comparison revealed a statistically significant difference between Group A and Group B; b pairwise comparison revealed a statistically significant difference between Group B and Group C; post hoc Tukey test was performed to test the significance of pairwise differences.

The percentage of women who had oocyte retrieval was 84.5% in Group A, 70% in Group B, and 80.3% in Group C. In terms of cycle cancellation rate, there were no significant differences between the three groups (P = 0.104).

There were significant differences between groups with and without successful ovarian stimulation allowing for the oocyte pick-up procedure stage in terms of age, body mass index (BMI), duration of infertility, progesterone level on the trigger day, number of follicles >17 mm on the trigger day, and endometrial thickness on the trigger day (P = 0.05) (Table 2). 

**Table 2:** Factors that influenced embryo transfer rates in women with DOR.

	OR (95% CI)	P-value
Age (years)	OR 0.97 (0.93–1.01)	0.092
BMI (kg/m2)	OR 0.97 (0.94–1.01)	0.113
Serum AMH (ng/mL)	OR 2.2 (0.7–7.2)	0.085
COH protocol		
Antagonist	OR 0.80 (0.50–1.20)	0.330
Agonist long protocol	OR 2.10 (1.10–4.30)	0.995
Microdose flare-up protocol	OR 1.1 (0.8–1.7)	0.125
Basal FSH (IU/L)	OR 0.98 (0.96–1.00)	0.524
Day 3 estradiol (pg/mL)	OR 0.99 (0.99–1.00)	0.107
Total AFC	OR 1.11 (0.93–1.25)	0.095
Duration of infertility (months)	OR 0.99 (0.99–1.00)	0.486
Estradiol on hCG day (pg/mL)	OR 1.00 (1.00–1.00)	0.001*
Progesterone on hCG day (pg/mL)	OR 0.97 (0.67–1.40)	0.124
Number of follicles >17 mm at hCG day	OR 1.46 (1.21–1.77)	0.001*
Endometrial thickness on the day of hCG (mm)	OR 1.08 (1.01–1.15)	0.001*

NS: Not significant; OR: odds ratio; CI: confidence interval; DOR: diminished ovarian reserve; BMI: body mass index; AMH: anti-Müllerian hormone; FSH: follicle-stimulating hormone; AFC: antral follicle count; hCG: human chorionic gonadotropin, *: P-values with statistical significance (P < 0.05).

The number of patients who had embryo transfer was 44 (52.4%) in Group A, 38 (46.9%) in Group B, and 119 (40.5%) in Group C. There were no significant differences between the three groups (P = 0.114). 

Univariate analysis revealed a significant relation between the embryo transfer rate and the peak E2 level, endometrial thickness, and number of follicles >17 mm on the day of HCG administration (Table 2).

Clinical pregnancy per transfer was 35.8% in Group A, 19.8% in Group B, and 29.5% in Group C, and there was no significant difference between the groups. All 3 groups had dual statistical comparison. Pairwise comparison did not reveal a statistically significant difference between Group A and Group B (P = 0.092), Group A and Group C (P = 0.141), or Group B and Group C (P = 0.874). These results might be due to the limited number of patients.

 Univariate analyses showed that age (OR: 0.89; 95% CI: 0.83–0.96) and basal FSH (OR: 0.93; 95% CI: 0.88–0.99) were negatively associated with clinical pregnancy rate (P = 0.05); on the other hand, AFC (OR: 1.14; 95% CI: 1.11–1.75), total number of oocytes retrieved (OR: 1.14; 95% CI: 1.03–1.26), number of mature oocytes (OR: 1.23; 95% CI: 1.10–1.38), and number of 2PN embryos (OR: 1.54; 95% CI: 1.26–1.87) were positively associated with clinical pregnancy rate (P = 0.05) (Table 3).

**Table 3:** Factors that influenced clinical pregnancy rates in women with DOR.

	OR (95% CI)	P-value
Age (years)	OR 0.89 (0.83–0.96)	0.050*
BMI (kg/m2)	OR 0.95 (0.88–1.02)	0.581
Serum AMH (ng/mL)	OR 10.2 (0.9–108.8)	0.454
COH protocol		
Antagonist	OR 0.80 (0.30–1.80)	0.261
Agonist long protocol	OR 1.80 (0.70–5.10)	0.474
Microdose flare-up protocol	OR 1.2 (0.6–1.8)	0.982
Basal FSH (IU/L)	OR 0.93 (0.88–0.99)	0.050*
Day 3 estradiol (pg/mL)	OR 0.99 (0.99–1.00)	0.231
Total AFC	OR 1.14 (1.11–1.75)	0.050*
Infertility duration (months)	OR 1.00 (0.99–1.00)	0.451
Estradiol on hCG day (pg/mL)	OR 1.00 (1.00–1.00)	0.362
Progesterone on hCG day (pg/mL)	OR 1.18 (0.66–2.09)	0.843
>17 mm follicle count	OR 1.07 (0.82–1.40)	0.274
Endometrial thickness on hCG day (mm)	OR 1.10 (0.99–1.23)	0.123
Total oocytes retrieved	OR 1.14 (1.03–1.26)	0.050*
Number of MII	OR 1.23 (1.10–1.38)	0.050*
Number of 2PN	OR 1.54(1.26-1.87)	0.050*
Number of transferred embryos	OR 1.64(1.36-1.97)	0.050*

NS: Not significant; OR: odds ratio; CI: confidence interval; DOR: diminished ovarian reserve; BMI: body mass index; AMH: anti-Müllerian hormone; FSH: follicle-stimulating hormone; AFC: antral follicle count; hCG: human chorionic gonadotropin; PN: pronuclear; MII: metaphase II, *: P-values with statistical significance (P < 0.05).

Multivariate logistic regression analysis showed that when the results were reanalyzed adjusting for age (Table 4), the woman’s age predicted the outcome of the treatment cycle (AUC = 0.634, P = 0.006), and the optimal cut-off value for age was found to be 37.5 years with 58% sensitivity and 54% specificity (Figure).

**Table 4:** Multivariate analysis model established for DOR patients using variables such as patient’s age, basal FSH levels, total AFC, and total oocytes retrieved.

Constant parameters	Unstandardized coefficients		Standardized coefficients beta	P
	B	SE		
Age (years)	–0.011	0.004	–0.156	0.003*
Basal FSH(IU/L)	–0.002	0.002	–0.055	0.316
Total AFC	0.020	0.010	0.104	0.055
Retrieved oocytes	0.016	0.007	0.136	0.015*

DOR: Diminished ovarian reserve; AFC: antral follicle count; FSH: follicle-stimulating hormone; B: standardized regression coefficient; SE: standard error; clinical pregnancy is the dependent variable. Enter model of regression was used for multivariate regression analysis.*: P-values with statistical significance (P < 0.05).

**Figure:**
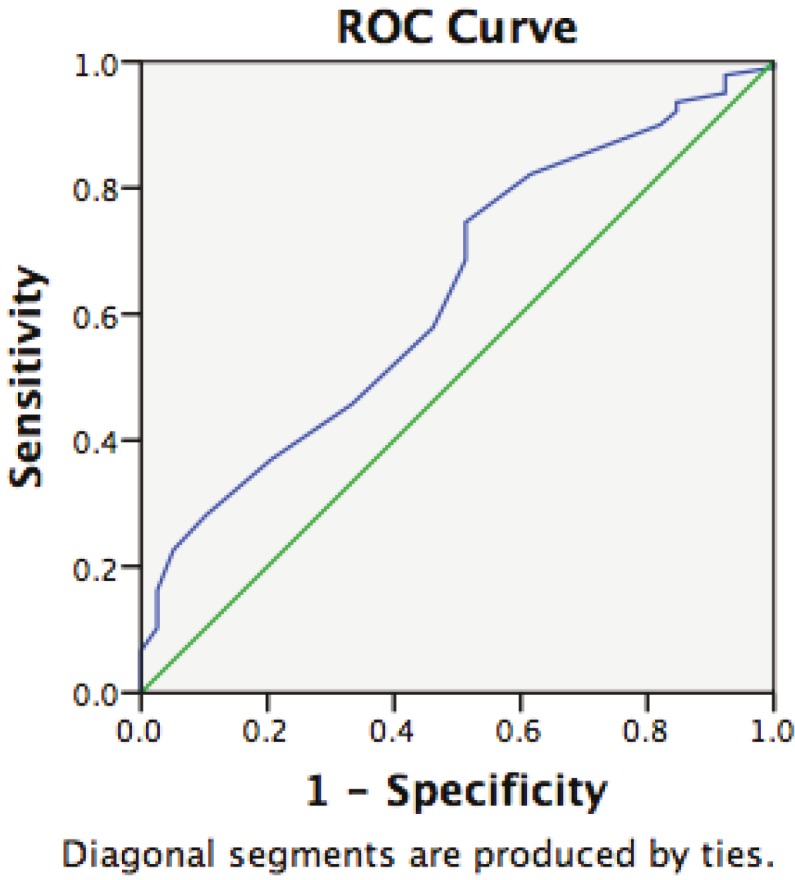
Accuracy of age and clinical pregnancy predictions. Receiver operating characteristic (ROC) curve analysis showed that the woman’s age predicted the outcome of the treatment cycle (AUC = 0.634, P = 0.006), and the optimal cut-off value for age was found to be 37.5 years with 58% sensitivity and 54% specificity.

## 4. Discussion

The purpose of the presented study was to draw attention to reduced ovarian reserve, which is the one of the most challenging issues of assisted reproduction. All DOR cases may have decreased ovarian reserve, but different causes for the DOR might have a different impact on the IVF/ICSI outcome. Our study showed that young women with DOR may have a higher possibility of clinical pregnancy than those with other causes.

Basal FSH and AFC are important markers of ovarian reserve, and the number of oocytes retrieved is a direct indicator of the ovarian reserve and the ovarian response to ovarian stimulation [7–9]. Some studies have suggested that elevated basal FSH concentrations are associated with impaired oocyte quality and decreased number of follicles that are ready to be stimulated [10,11]. Elevated basal FSH also affects the number of oocytes retrieved and the implantation rates after IVF/ICSI. In contrast to these findings, some studies demonstrated that high FSH levels had not lowered the implantation rate in patients with DOR, although pregnancy rates were found to be lower in comparison to the control group [7,12]. In our study, FSH concentrations in young DOR patients were relatively lower than in other groups and they had higher implantation and pregnancy rates than the other groups, but the differences did not reach statistical significance (P = 0.195 and P = 0.113, respectively). According to multivariable analysis, clinical pregnancy rates increased in DOR patients with lower FSH concentrations and higher antral follicle counts.

Female fecundity declines with age due to decreased ovarian reserve, but reduced ovarian reserve can be encountered occasionally in younger women [13,14] due to the fact that biological aging of the ovaries can occur independent of chronological age [15].

Several studies [16–18] investigated the predictive value of the number of oocytes retrieved for the pregnancy rate in IVF-ET cycles. Authors claimed that there is an optimal range of oocytes retrieved for achieving pregnancy. Van der Gaast et al. [18] demonstrated a clear correlation between pregnancy rate and the number of oocytes retrieved in poor responders. Consistent with these studies, we found that a higher number of follicles with a diameter of >17 mm on the day of hCG administration increases the embryo transfer rates and the total number of oocytes retrieved from DOR patients, resulting in increased clinical pregnancy rates.

Interestingly, the number of mature oocytes and number of total oocytes retrieved from younger women were found to be lower, but their clinical pregnancy rates were found to be slightly higher than those of older women with DOR. It can be speculated that this result might be related to the use of more modest stimulation protocols, as previously suggested by Silber et al. [19]. The results of the present study are similar to those of Kumbak et al.’s research [7], which reported a lower mature (M2) oocyte rate in the young DOR group. 

Ovarian surgery is another cause of DOR. Endometrioma is one of the most frequent pathologies in gynecological surgery. Despite the potential negative effect of ovarian cystectomy on ovarian reserve and future fertility, surgical resection is the preferred treatment in cases with serious pain symptoms or where it is necessary to rule out the possibility of malignancy. Several mechanisms have been described to explain this quantitative alteration of ovarian reserve after cystectomy: accidental removal of follicle-bearing healthy ovarian tissue adherent to the cyst wall during cystectomy, vascular compromise due to electrosurgical coagulation, local inflammation, and autoimmune reactions. 

Guler et al. reported that cycle cancellation was significantly higher and clinical pregnancy per cycle and per embryo transfer was significantly lower in the operated endometrioma group in comparison to patients with intact endometriomas, and they concluded that laparoscopic endometrioma cystectomy may worsen IVF/ICSI outcomes by decreasing ovarian reserve and the quality of oocytes and embryos [20].

Roustan et al. compared IVF live birth rates in patients with DOR following cystectomy for endometrioma with patients with idiopathic DOR and reported a significantly higher fertilization rate and mean number of embryos but a reduced clinical pregnancy rate in women who were previously operated on for endometrioma [5].The authors concluded that surgical removal of endometriomas had no beneficial effect on IVF results. 

Although we observed a trend for a higher mean number of retrieved oocytes and mature oocytes in women with DOR who had cystectomy for endometrioma, fertilization rate, number of top quality embryos, and clinical pregnancy rates were found to be lower that in young women with DOR. Other studies [21,22] reported a significantly reduced clinical pregnancy rate and higher IVF cancellation rate in women who were previously operated on for endometriomas. In our study, despite the higher ICSI cancellation rate, the clinical pregnancy rate was found to be higher in the idiopathic DOR group compared to the previous ovarian surgery group. 

The major limitation of the present study is the retrospective case-control design. 

In conclusion, although response to ovarian stimulation, clinical pregnancy, and live birth rates were not significantly different in women with DOR related to different etiological factors, young women with DOR may benefit from ART as the pregnancy rates are encouraging. This should be kept in mind while counseling and managing young DOR patients receiving IVF/ICSI treatment.
